# The stunting syndrome in developing countries

**DOI:** 10.1179/2046905514Y.0000000158

**Published:** 2014-04

**Authors:** Andrew J Prendergast, Jean H Humphrey

**Affiliations:** 1 Centre for Paediatrics, Blizard Institute, Queen Mary University of London, UK; 2 Zvitambo Institute for Maternal and Child Health Research, Harare, Zimbabwe; 3 Department of International Health, Johns Hopkins Bloomberg School of Public Health, Baltimore, MD, USA

**Keywords:** Stunting, Malnutrition, Mortality, Neurodevelopment, Infections

## Abstract

Linear growth failure is the most common form of undernutrition globally. With an estimated 165 million children below 5 years of age affected, stunting has been identified as a major public health priority, and there are ambitious targets to reduce the prevalence of stunting by 40% between 2010 and 2025. We view this condition as a ‘stunting syndrome’ in which multiple pathological changes marked by linear growth retardation in early life are associated with increased morbidity and mortality, reduced physical, neurodevelopmental and economic capacity and an elevated risk of metabolic disease into adulthood. Stunting is a cyclical process because women who were themselves stunted in childhood tend to have stunted offspring, creating an intergenerational cycle of poverty and reduced human capital that is difficult to break. In this review, the mechanisms underlying linear growth failure at different ages are described, the short-, medium- and long-term consequences of stunting are discussed, and the evidence for windows of opportunity during the life cycle to target interventions at the stunting syndrome are evaluated.

## INTRODUCTION

Linear growth failure in childhood is the most prevalent form of undernutrition globally.^1^ An estimated 165 million children under 5 years of age are *stunted*, with a height-for-age Z-score (HAZ) below −2 (i.e. more than two standard deviations below the population median), but a larger number of children with HAZ >−2 still have inadequate linear growth and are therefore experiencing *stunting*.^1^ Undernutrition underlies 45% of all child deaths among children <5 years,^2^ although mortality has been described as the ‘tip of the iceberg’ of malnutrition.^3^ Stunting more pervasively hinders developmental potential and human capital of entire societies due to its longer-term impact on cognitive function and adult economic productivity; it is therefore considered the best surrogate marker of child health inequalities.^4^,^5^

After many years of neglect, stunting has now been identified as a major global health priority.^6^ Ambitious World Health Assembly targets aim to reduce stunting by 40% between 2010 and 2025.^7^ Whilst impressive progress has been made in Asia, with a decline in the proportion of stunted children from 49% to 28% between 1990 and 2010, this is still the continent with the most stunted children globally (approximately 100 million); in Africa, stunting prevalence has remained stagnant around 40% and, owing to population growth, the absolute number of stunted children is increasing.^8^ Millennium Development Goal 1 (MDG 1), which focuses on eradicating extreme hunger and poverty, uses underweight rather than stunting as its target (http://www.un.org/millenniumgoals/). Weight is more frequently used to monitor growth than is height, because of ease of measurement; however, underweight (low weight-for-age) does not distinguish between stunting (low height-for-age) and wasting (low weight-for-height). In settings where stunting is highly prevalent and wasting is rare, underweight therefore underestimates the burden of malnutrition.^9^–^11^ Overall, developing countries have <5% chance of achieving MDG 1 (reducing the proportion of children with WAZ<−2 by half between 1990 and 2015), although 61 of 141 countries are estimated to have a 50–100% chance of success.^12^ Even regions such as the Caribbean and Latin America which are on target to reach MDG 1 when measured as WAZ would not achieve this target if using HAZ as the indicator.^13^

Although usually described separately, stunting, underweight and wasting frequently co-exist and children with multiple measures of anthropometric failure have a compounded risk of morbidity and mortality.^14^,^15^ For example, analysis of data on 53,767 children in Africa, Asia and Latin America demonstrated that mortality in those who were stunted and underweight was more than three times greater than in well nourished children [HR 3.4 (95% CI 2.6–4.3)]; this risk rose to >12-fold [HR 12.3 (7.7- 19.6)] in children who were stunted, underweight and wasted.^15^ Thus, although stunting and wasting have tended to be viewed separately, there is a growing impetus to consider both conditions together.^16^ Weight-for-height tends to reflect more short-term inadequacy of dietary intake or utilization; the effectiveness of therapeutic feeding for wasted children is well established.^17^ By contrast, the mechanisms underlying linear growth failure and interventions to prevent or ameliorate stunting are less clearly defined.^16^

## THE STUNTING SYNDROME

**Figure 1:**
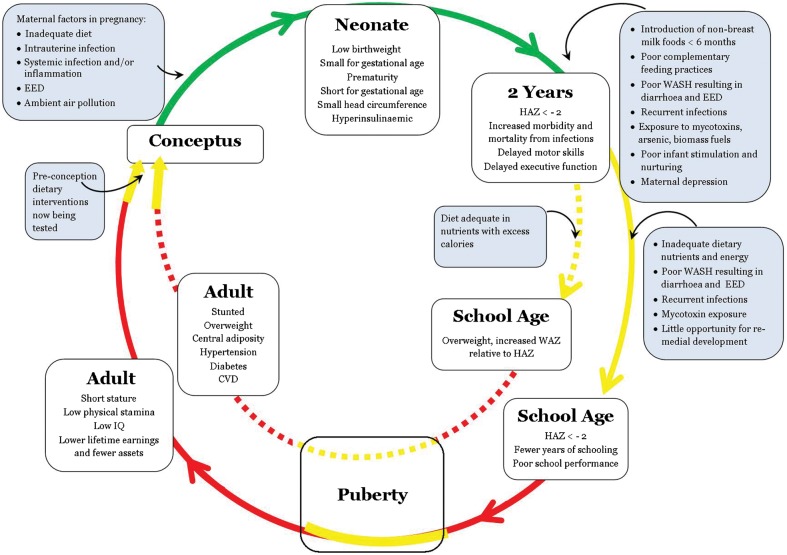
The stunting syndrome. The green pathway denotes the period between conception and 2 years (‘*the first 1000 days*’) when stunting and probably all associated pathology are most responsive to, or preventable by, interventions. The yellow pathway denotes periods between age 2 years and mid-childhood and during the adolescent growth spurt when some catch-up in linear growth may occur, though effects during these periods on other components of the stunting syndrome (e.g. cognition and immune function) are less clear. The short yellow pathway before *Conceptus* reflects evidence that dietary interventions targeting stunted women during the pre-conception period improve birth outcomes. The red pathway denotes periods when the stunting syndrome appears unresponsive to interventions. Blue boxes list age-specific causative or aggravating factors. White boxes describe common age-specific outcomes. Between 2 years and adulthood, the pathways diverge to denote: dashed line, a stunted child whose environment becomes more affluent with abundant access to food, causing excessive weight gain; solid line: a stunted child whose environment remains resource-constrained/food insecure. Please see the text for citations

Although stunted children are identified by comparing their height to an age- and sex-matched reference population, short stature is not usually in itself problematic. Instead, we view this condition as a ‘stunting syndrome’ in which multiple pathological changes marked by linear growth retardation increase morbidity and mortality and reduce physical, neurodevelopmental and economic capacity. The short-, medium- and long-term sequelae of stunting, summarised in Figure 1, are discussed in detail later. Stunting is a cyclical process because women who were themselves stunted in childhood tend to have stunted offspring, creating an intergenerational cycle of poverty and reduced human capital that is difficult to break,^18^ although potential windows of opportunity have been identified (Fig. 1).

## DETERMINANTS OF HEALTHY GROWTH

There are four interlinked phases of growth during the life-course: fetal, infant, childhood and pubertal, each governed by different regulatory mechanisms. A study of healthy children who were measured frequently during the first 21 months of life demonstrated that growth is an episodic phenomenon, with long periods of stasis (between 2 and 63 days) punctuated by short phases of saltation (growth spurts), so that there is no growth during 90–95% of healthy infancy.^19^ Although height is a highly heritable trait, with over 200 genes identified in genome-wide association studies, together they explain only about 10% of the variation in adult height.^20^,^21^ Children from different geographical areas grow at a remarkably similar rate during fetal life^22^ and the first few years of postnatal life^23^–^25^ if born to mothers whose nutritional and health needs are met and if raised in unconstrained conditions. Environmental factors such as maternal nutritional status, feeding practices, hygiene and sanitation, frequency of infections and access to healthcare are therefore the major determinants of growth in the first 2 years of life.^18^

Studies of variation in stunting prevalence across countries^26^ and between different populations within countries^27^,^28^ demonstrate the importance of socio-economic factors. Stunting prevalence is therefore a good indicator of inequalities in human development;^29^ across 80 countries, those with a high prevalence of stunting tended also to have larger socio-economic inequalities in children with stunting.^30^ An analysis for the Lancet Nutrition Series incorporating data from 79 countries showed that stunting prevalence was 2.47 (range 1.00–7.64) times higher in the poorest than in the richest quintile.^2^ In China, where there was marked economic development between 1975 and 2010, there was a parallel improvement in the growth of children and adolescents; height showed a strong positive correlation with GDP per capita (*r* 0.90, *P*<0.0001) and with urbanisation (*r* 0.92, *P*<0.0001), and a negative correlation with the under-5 mortality rate (*r* −0.95, *P*<0.0001).^31^ Using data from 7630 mother–child pairs enrolled in the COHORTS study in Brazil, Guatemala, Philippines, India and South Africa, children on average had better heights than their mothers owing to secular trends arising from improvements in economic and environmental conditions over time.^32^ Improvements in growth are seen when people migrate from poor nations to countries with better socio-economic conditions.^18^

## THE TIMING OF GROWTH FALTERING

Stunting begins *in utero* and continues for at least the first 2 years of postnatal life; the period from conception to a child’s second birthday (the first thousand days) has therefore been identified as the most critical window of opportunity for interventions (www.thousanddays.org). The average length-for-age Z-score among newborns in developing countries is approximately −0.5 and continues to decline after birth to reach a nadir of around −2.0 by 18–24 months of age.^33^ Upon publication of new WHO growth standards in 2006,^34^ it became apparent that linear growth faltering in developing countries occurs sooner after birth that had been appreciated using prior National Center for Health Statistics (NCHS) data,^35^ which were based on the growth of predominantly formula-fed infants.^36^ Although stunting at a population level follows the trajectory described above, there are widespread between-child differences in stunting patterns when individual child growth curves are examined (Prendergast *et al.*, unpublished data). There are some difficulties in interpreting the patterns of long-term linear growth from published data, because many studies are cross-sectional rather than longitudinal; most focus on children <5 years of age; and height is generally presented as Z-scores, which can be problematic when used to describe growth over time, as discussed later. Across multiple countries in sub-Saharan Africa it has been shown that boys are more likely to be stunted than girls (odds ratio 1.16, 95% CI 1.12–1.20) for reasons which remain unclear.^37^

## THE PATHOGENESIS OF STUNTING

Despite the high global prevalence of stunting, the pathogenesis underlying linear growth failure is surprisingly poorly understood. For this reason, the most tractable pathways for effective interventions to promote healthy growth remain unclear,^38^ and no research study has ever normalised linear growth among children living in developing countries. From epidemiological studies it is apparent that suboptimal breastfeeding and complementary feeding practices, recurrent infections and micronutrient deficiencies are important proximal determinants of stunting.^2^,^39^ Linear growth failure also occurs within a complex interplay of more distal community and societal factors, such as access to healthcare and education, political stability, urbanisation, population density and social support networks, which have been captured in the WHO Conceptual Framework on Childhood Stunting, as recently reviewed.^40^ Below we review the current understanding of growth failure across the life-course and try to identify potential windows of opportunity for interventions (Fig. 1).

### Antenatal period

Fetal growth is governed by complex interactions between maternal nutritional status, endocrine and metabolic signals and placental development. Newborn size is therefore a reflection of the intrauterine environment; prevalence of low birthweight (<2.5 kg) is approximately six times higher in developing compared to developed countries.^41^ The INTERGROWTH-21st Project, a population-based study of fetal growth across eight countries, showed that newborn length was very similar across sites among affluent, healthy, educated women.^22^ Low birthweight babies include those born too soon (preterm), too small (small for gestational age, SGA), or both. In 2010, 27% of liveborn infants globally were SGA and almost 3 million infants were born preterm and SGA;^42^ risks of poor growth^43^ and mortality^44^ are compounded among those born preterm-SGA. Using data from 19 birth cohorts, Christian *et al.* showed that, relative to infants born appropriate for gestational age and at term, the risk of postnatal stunting increased markedly among infants born preterm [OR 1.93 (95% CI 1.71–2.18)], SGA [2.43 (2.22–2.66)] and SGA-preterm [4.51 (3.42–5.93)].^43^ Overall, they estimated that 20% of stunting has *in utero* origins. In some settings, antenatal determinants of stunting appear to be even more important than postnatal determinants.^45^,^46^

Maternal undernutrition contributes to an estimated 20% of maternal deaths and increases the risk of adverse pregnancy outcomes, childhood mortality and stunting.^47^ Short maternal stature, low body mass index and poor weight gain during pregnancy are the major indices associated with low birthweight.^41^,^48^ Early pregnancy during adolescence, when mothers are themselves still growing, increases the risk of further maternal stunting^49^,^50^ and leads to adverse obstetric outcomes.^51^,^52^ Closely spaced births also increase nutritional demands on the mother.^53^ Maternal height is associated with size at birth and with postnatal stunting,^32^,^54^–^57^ which compounds the intergenerational cycle of stunting (Fig. 1). Birthweight and length are themselves related to subsequent growth in childhood. A longitudinal analysis of data from five birth cohorts^32^ which used a measure of conditional height across the life-course to account for the colinearity of multiple growth measures showed that maternal height was related to offspring height at all ages (correlations ranging from 0.15 to 0.55, *P*<0.001) and maternal height was strongly associated with stunting prevalence at 2 years, similar to findings from previous cross-sectional studies.^54^,^56^,^57^ The largest study to date^55^ incorporating data from 109 demographic and health surveys across 54 countries showed that maternal height was inversely associated with mortality, underweight and stunting during infancy and childhood.

Given these strong intergenerational effects, the concept of stunted families rather than stunted children has been proposed,^58^ particularly since paternal nutritional status can also influence childhood stunting.^59^ It has been argued that these intergenerational influences on health have been ignored in setting the Millennium Development Goals, which are unlikely to be attained if maternal health is not addressed.^60^ Since infants are entirely dependent on their mother for nutrition during the first 500 days of life,^61^ several trials have focused on improving maternal nutritional status. Prenatal multiple micronutrients^62^ and provision of balanced energy and protein to mothers^63^ reduce SGA by 9% and 31%, respectively. Daily iron supplementation during pregnancy reduces low birthweight by 20%^64^ but zinc supplementation has no significant effect on birthweight.^65^ Calcium supplementation in pregnant women increases birthweight by 85 g (95% CI 37–133) compared with controls,^66^ and three trials of vitamin D in pregnancy showed a borderline significant effect on low birthweight (relative risk 0.48, 95% CI 0.23–1.01).^67^

Various human and animal studies have shown that maternal diet can mediate epigenetic changes in the fetus.^68^–^70^ In a Gambian trial of multiple micronutrient supplementation during the peri-conception period, epigenetic changes were seen in several differentially methylated regions which govern growth or immune function.^71^,^72^ Prenatal interventions can therefore have an impact on postnatal growth,^73^–^76^ as was shown in the MINIMAT trial in Bangladesh^76^ in which early food supplementation during pregnancy reduced postnatal stunting in boys (but not girls), consistent with a fetal programming effect; however, not all antenatal interventions have demonstrated a long-term effect.^73^,^77^,^78^

### Birth to 6 months

Healthy infants experience maximal growth velocity between birth and 6 months of age. Furthermore, the first few months of life appear particularly critical for long-term neurodevelopment.^79^ When linear growth among infants in developing countries was evaluated using 2006 WHO growth standards, the prevalence of stunting in the first half of infancy doubled compared to prior estimates;^80^ in some countries, such as India, 20% of infants are stunted before 6 months of age.^81^,^82^ There is therefore an increasing appreciation of undernutrition in infants under 6 months, who are often excluded from nutrition surveys and marginalised in nutrition programmes,^83^ and a realisation that interventions need to be targeted earlier than was previously appreciated.

Exclusive breastfeeding (EBF) for the first 6 months has been recommended by WHO since 2001.^84^ Although the benefits of EBF for reduced morbidity and mortality^47^ and improved cognition^85^ are clear, evidence for an effect on linear growth is surprisingly weak.^86^ Promotion of EBF is effective at increasing EBF rates,^86^ but in a cluster-randomised trial of EBF promotion in three African countries, increased rates of EBF were not associated with improved HAZ scores at 6 months.^87^ In a secondary data analysis of national survey data from India, even among infants categorized as practicing EBF, stunting prevalence was 21% by 6 months, although the definition of EBF was based only on a 24-hour recall period.^81^ Growth failure that is continuous from fetal life through the first 6 months of postnatal life suggests the existence of common factors, which have not been well defined.^88^ In a study of Zimbabwean mother–infant pairs, there was evidence of chronic inflammation very early in life (by 6 weeks of age). Levels of inflammatory markers (e.g. CRP) were persistently higher in stunted than in non-stunted infants, and were associated with the level of maternal inflammation at birth, suggesting one potential common mechanism linking antenatal and postnatal growth failure.^89^

### 6–24 months of age

The period from 6 to 24 months of age is one of the most critical periods for linear growth;^90^ it is also the time of peak stunting prevalence in developing countries, due to high demand for nutrients coupled with limited quality and quantity of complementary foods.^91^ Complementary feeding refers to the timely introduction of safe and nutritious foods in addition to continued breastfeeding;^92^ the majority of stunting interventions have been targeted at improving infant and young child feeding (IYCF) practices, which are known to be poor in many settings.^93^ WHO has developed a set of IYCF indicators to evaluate feeding practices; although in some studies these show associations with stunting,^94^ in others they have been found to lack sensitivity and specificity.^95^

Interventions to improve IYCF have generally focused on nutrition counselling, providing complementary food with or without micronutrients, and increasing energy density of complementary foods through simple technology.^91^ A meta-analysis of 42 studies showed a modest impact of complementary feeding interventions; the best improvement in linear growth was around +0.7 gain in HAZ, which is around one-third of the average deficit.^91^ Generally, studies providing complementary foods in food-insecure regions showed some benefit; micronutrient fortification alone showed little or no impact on growth. Complementary feeding education improves linear growth, and leads to declines in stunting among food-insecure populations, but effect sizes are modest.^96^ Community-based supplementary feeding (i.e. giving additional food beyond the normal home diet) has a limited evidence base; the only impact on length was seen in children below 12 months of age in two trials.^97^

Lipid-based nutrient supplements (LNS), which are micronutrient-fortified ready-to-use products, have been used during the complementary feeding period in several trials. LNS showed a significant impact on linear growth in studies from Ghana,^98^ Congo^99^ and Haiti^100^ but not in two trials from Malawi.^101^,^102^ Another Malawian study^103^ showed an impact of LNS on severe stunting when provided to 6-month-old infants for 1 year, but not during a short 12 week intervention;^104^ the effect was sustained up to 3 years of age,^105^ although, of note, there was no control group in these trials. Taken together, despite differences in treatment duration, comparator groups, primary outcomes (use of absolute HAZ score *vs* proportion stunted) and length of follow-up, there is some evidence from trials for a small but measurable effect of LNS on linear growth.

Deficiencies of vitamin A, zinc, iron and iodine are common,^47^ and multiple micronutrient deficiencies are often found in the same child,^106^ affecting many aspects of physiology, including neural and immune function. Globally, two billion people are at risk of micronutrient deficiency, which has been termed ‘hidden hunger’ due to its impact on health and human capital.^107^ A systematic review of micronutrient powders^108^ found relatively few studies which evaluated growth; none suggested benefit and there was increased diarrhoea in some children taking micronutrient powders. An estimated 17.3% (95% CI 15.9–18.8) of the global population is at risk of zinc deficiency^2^ and country-specific prevalence of inadequate zinc intake correlated with stunting prevalence in 138 low- and middle-income countries (*r* 0.48, *P*<0.001).^109^ Daily zinc (10 mg/day) for 24 weeks leads to a mean (SD) height gain of approximately 0.38 cm (0.25).^110^ Studies investigating the impact of vitamin A on growth have reported mixed results.^111^–^114^

Several decades ago, detailed longitudinal studies in Guatemala showed that recurrent infections can impair growth, particularly during the second half of infancy;^115^ subsequent studies have confirmed this observation.^90^,^116^–^119^ However, there is a bidirectional relationship between infections and malnutrition; several studies show that malnourished children have an increased frequency, duration and severity of infections.^116^,^120^–^123^ It can therefore be difficult to distinguish cause and effect, although animal models provide a useful system to explore these interactions. Mice experimentally infected with *Cryptosporidium*, entero-aggregative *Escherichia coli* or *Giardia lamblia* develop enteropathy and growth impairment; when they are malnourished, they have a higher pathogen load and more severe enteropathy.^124^–^127^

Stunting and parasitic infections overlap geographically, and several studies have explored associations between them. Studies conducted over a 25-year period variously reported that malaria was associated with an increased risk of stunting,^128^–^132^ a decreased risk^133^ or no association;^134^–^137^ methodological differences, confounding and the possibility of reverse causality made it difficult to interpret these conflicting findings. A study that used Mendelian randomisation to explore associations between malaria and stunting has helped to clarify these associations.^138^ Children with sickle cell trait are asymptomatic but protected from malaria; using matching to control for unmeasured confounding, the authors showed a causal effect of malaria on stunting, since sickle cell trait can only influence stunting through its effect on malaria. Intestinal helminths affect two billion people worldwide and may affect nutritional status through reduced digestion and absorption, chronic inflammation and loss of nutrients. However, as for malaria, the impact of helminths on stunting is difficult to assess because of confounding and the possibility of reverse causality in published studies.^139^–^140^ Polyparasitism is common and is associated with adverse child health outcomes;^141^–^143^ there is a growing appreciation of the burden of helminths in pre-school children^144^–^147^ in whom infection may have the greatest impact on growth and development.^148^

Diarrhoea is one of the most frequent infections in childhood, particularly in conditions of poor sanitation and hygiene, although early studies varied in their conclusions as to whether diarrhoea did^117^,^149^,^150^ or did not^116^,^151^ affect stunting. Effects of diarrhoea may be short term^152^ because catch-up growth can occur between episodes.^153^ In an analysis of data from nine community-based studies with daily diarrhoea data and longitudinal anthopometric measurements, the odds of stunting by 24 months increased multiplicatively with each episode of diarrhoea; overall, 25% of stunting was attributed to five or more episodes of diarrhoea.^154^ In a more recent study of seven longitudinal infant cohorts, cumulative diarrhoeal burden had a smaller but measurable effect on linear growth; a child with the typical diarrhoea burden (equivalent to 23 days per year) was 0.38 cm shorter at 2 years of age than a child without diarrhoea.^155^

It is increasingly apparent that subclinical infection with enteric pathogens is common, even in the absence of diarrhoea.^156^ In conditions of poverty, where children experience frequent exposure to enteric pathogens due to faeco-oral transmission, a population shift in gut structure and function occurs.^157^,^158^ This subclinical pathology, characterised by villous atrophy and chronic inflammation of the small intestine, has been termed environmental enteric dysfunction (EED), and is associated with modest malabsorption and increased intestinal permeability.^159^ Even mild malabsorption may be important in the context of a marginal diet and rapid growth, and permeability enables translocation of microbial products from the lumen of the gut to the systemic circulation, where they can trigger chronic inflammation, which suppresses IGF-1.^89^,^160^ Early studies from The Gambia^161^–^163^ and elsewhere^164^,^165^ confirmed that abnormal intestinal permeability is common; linear growth in Gambian infants was inversely related to permeability.^166^ More recent studies^89^,^167^–^169^ and reviews^159^,^160^,^170^,^171^ have refocused attention on the plausible links between gut inflammation and stunting; given its chronic nature, it has been argued that EED may be a more important pathway to stunting than is diarrhoea.^160^ We therefore view stunting as an inflammatory disease arising, in part, from primary gut pathology. Since gut damage also occurs with recurrent (especially persistent) diarrhoea, severe acute malnutrition, HIV infection and micronutrient deficiencies, there are multiple overlapping causes of enteropathy in settings of poverty which may exacerbate the growth failure arising from EED.^171^

In the last few years, studies using next-generation sequencing have begun to characterise the composition and function of the intestinal microbiota during malnutrition (recently reviewed by Ahmed *et al.*^172^). Assembly of the microbiota occurs over the first 3 years of life,^173^ with a founding population acquired from the mother and subsequent composition shaped by environmental influences, including the diet.^174^–^176^ The microbiota has important roles in metabolism of macro- and micronutrients, immune development, integrity of the intestinal barrier and defence against enteric pathogens.^172^ A study of Malawian twin pairs discordant for kwashiorkor highlighted the importance of the gut microbiome (the genes expressed by the microbiota) in malnutrition.^177^ Kwashiorkor was characterised by a functionally immature microbiome, which matured during therapeutic feeding (with enrichment of potentially beneficial organisms, such as Bifidobacteria and Lactobacillus) but was not sustained once therapeutic feeding was stopped. In a subsequent study from Bangladesh,^178^ sequencing of monthly faecal samples from 50 children with healthy growth enabled ‘microbiota-for-age’ Z-scores to be developed. Children with acute malnutrition showed immaturity of their microbiota, and the microbiota-for-age Z score correlated with weight-for-height Z-score at 18 months of age. Whilst these studies provide emerging evidence for a role of the microbiota in malnutrition, no published studies to date have characterised the microbiota of children with stunting.

Recurrent infections, inflammation and gut damage are all potentially amenable to interventions. A meta-analysis of ten trials from low- and middle-income countries showed that antibiotics had a significant impact on both weight and height gain; although antibiotics were given for a range of indications (not specifically for growth), a side-benefit may have been growth-promoting modulation of the intestinal microbiota and/or resolution of subclinical infections.^179^ There is current interest in azithromycin, a broad-spectrum, long-acting, immunomodulatory antibiotic that is commonly used in mass drug administration programmes for trachoma and improves child survival.^180^ However, a comparison of one versus two doses of azithromycin in Niger showed no difference in height between groups, although the study was cross-sectional and underpowered for this outcome;^181^ other trials are underway. A trial of rifaximin, a non-absorbable broad-spectrum antibiotic, among 3–5 year old children in Malawi was designed to test the hypothesis that a short course of antibiotics would reduce EED.^182^ At baseline and after 7 days of rifaximin, children were administered a combination of lactulose and mannitol, and researchers measured the ratio of the two sugars in the urine (L:M ratio), which provides a measure of small intestinal absorption and permeability. Although changes in L:M (the primary endpoint) were similar between rifaximin and placebo groups, leading the authors to speculate that bacterial overgrowth was not important, the trial assessed only one domain of EED,^159^ and the treatment duration was short.^182^ The same researchers undertook a three-arm randomised trial among 1–3 year old asymptomatic Malawian children, comparing single-dose albendazole, 14 days of zinc, or placebo; increases in L:M ratio were greater in the placebo compared to intervention groups, providing some promise that a short intervention may modulate markers of EED, although growth was not assessed.^183^ Several studies have evaluated the impact of probiotics on growth at different ages; overall, results are conflicting due to differences in duration, strain and dose of probiotics,^184^ but some have suggested benefit, mostly in weight gain.^185^–^188^ A trial of probiotics (*Lactobacillus* GG) in Malawian children at risk of EED showed no difference in L:M ratio after 30 days of treatment compared to placebo.^189^ The first study to evaluate an immunomodulatory approach to EED randomised children with severe acute malnutrition to 28 days of mesalazine (an aminosalicylate) or placebo during nutritional rehabilitation; although designed as a safety study with a primary outcome of adverse events (which were similar between groups), there was a trend towards reduction in several systemic inflammatory markers, suggesting that anti-inflammatory interventions are safe and warrant further evaluation in larger trials including growth outcomes.^190^

Millennium Development Goal 7c aims to halve the proportion of the population without sustainable access to safe drinking water and basic sanitation by 2015. The benefits of improved water, sanitation and hygiene (WASH) have mostly been evaluated in terms of reducing diarrhoea^191^ and soil-transmitted helminth infections,^192^ but it has been argued that the potential impact of WASH on EED and stunting has been undervalued.^160^ Observational studies support an association between WASH conditions and child height,^168^,^193^–^199^ and a recent meta-analysis of five cluster-randomised controlled trials which evaluated water disinfection, provision of soap or improvement in water quality (although no trials of improved sanitation) showed a small but significant impact on HAZ (mean difference 0.08, 95% CI 0.00–0.16).^200^ It has also been argued that WASH has potential to improve early child development through effects on inflammation, anaemia and stunting.^201^ Interventions need to be targeted at the pathways through which faeco-oral transmission commonly occurs in infancy, such as exploratory behaviour leading to ingestion of animal faeces and soil,^202^ known to be highly contaminated in settings where people live with livestock;^203^,^204^ this infant-targeted approach has been termed ‘baby WASH’ [201]. Cluster-randomised trials are currently evaluating the impact of improved WASH on stunting in Zimbabwe (clinicaltrials.gov identifier NCT01824940), Kenya (NCT01704105) and Bangladesh (NCT01590095).

There are observational data supporting associations between other environmental exposures and stunting. Mycotoxins are fungal metabolites that frequently contaminate staple foods such as maize and ground nuts in developing countries; infants tend to be particularly vulnerable to exposure in the food chain.^205^ Levels of aflatoxin were related to stunting in a cross-sectional study of children aged 9 months to 5 years in Benin and Togo^206^ and in Tanzania, exposure to fumonisin was inversely associated with linear growth in infancy.^207^ Mycotoxins might mediate stunting through multiple pathways, including enteropathy, although further studies are needed to clarify causation.^208^ Arsenic exposure during pregnancy has been linked to low birthweight^209^ and urinary arsenic metabolites were inversely related to plasma IGF-1 levels in Bangladeshi pre-school children.^210^ Half the global population uses biomass fuels such as dung, coal, charcoal or wood, which give rise to indoor pollution. In some studies,^211^,^212^ but not others,^213^ exposure to biomass fuels was associated with stunting in children, after adjusting for covariates.

### Beyond 24 months of age

Stunting has tended to be viewed as a condition determined in the first 1000 days, because linear growth failure begins antenatally^43^ and continues over the first 24 months, with little apparent recovery thereafter.^33^ However, recently it has been proposed that the window of opportunity for catch-up growth may extend beyond 24 months, using longitudinal data from the COHORTS study,^214^ from rural Gambia,^214^ and from the Young Lives study in Ethiopia, Peru, India and Vietnam.^215^–^217^ However, there has been debate about the use of height-for-age Z-scores to describe changes in growth over time, because HAZ calculations include the age- and sex-specific standard deviation for height as the denominator, which increases with age; for a constant absolute height deficit, therefore, the HAZ will tend to become less negative over time, leading to apparent recovery in linear growth.^218^ This was well demonstrated in a recent analysis in which HAZ among children from three birth cohorts (South Africa, Guatemala, The Philippines) appeared to improve from 24 months to mid-childhood whilst the absolute height deficit continued to accumulate.^219^ Leroy *et al.* in a comparison of absolute height-for-age differences (HAD) with HAZ in 51 nationwide surveys showed that whilst HAZ appeared to level off between 24 and 60 months, HAD continued to increase; 70% of the shortfall in height occurred in the first 1000 days and 30% between 2 and 5 years of age.^220^

The question of potential recovery beyond the first 1000 days remains important and windows of opportunity for different components of the stunting syndrome may open and close at different times (Fig. 1). What causes ongoing linear growth faltering beyond 24 months, and whether interventions would usefully improve lean mass rather than increasing the risk of long-term obesity, remain uncertain.^220^ Adolescence is the time beyond infancy when growth velocity is maximal and represents the last opportunity for catch-up growth,^221^ although to achieve full growth potential probably requires intergenerational catch-up growth.^222^

## CONSEQUENCES OF THE STUNTING SYNDROME

Short stature is an easily measurable indicator of a syndrome that has far-reaching consequences across the life-course (Fig. 1).

### Morbidity and mortality

Short-term, stunting is associated with increased morbidity and mortality from infections, in particular pneumonia and diarrhoea.^47^,^223^–^225^ In a recent large analysis including individual-level data from 10 studies in Asia, Africa and South America, there was a clear dose-response relationship between HAZ and mortality, although not all datasets had information on potential confounders.^224^ Even children who were stunting but not stunted (HAZ between −2 and <−1) had an elevated risk of respiratory infections (HR 1.55, 95% CI 1.02–2.37) and diarrhoea (HR 1.67, 95% CI 1.20–2.30); children who were severely stunted (HAZ <−3) had a much greater risk (HR 6.39, 95% CI 4.19–9.75 and 6.33, 4.64–8.65, respectively). Severely stunted children also had a three-fold increased risk of mortality from other infections including sepsis, meningitis, tuberculosis, hepatitis and cellulitis (HR 3.01, 95% CI 1.55–5.82), suggesting a generalised immune defect in children with poor linear growth.

Although undernutrition is the commonest global immunodeficiency, the specific immune defects associated with stunting have not been well characterised. A recent systematic review^226^ summarised the literature on immune function in malnutrition but highlighted that most are cross-sectional studies of hospitalised children with SAM, using varied definitions of malnutrition, and conducted several decades ago. Malnourished children have complex derangements in physiology, impaired mucosal integrity, poor macro- and micro-nutrient status and multiple co-infections, and picking apart these contributory factors is challenging. Undernutrition appears to affect both innate and adaptive immunity,^226^ but more carefully designed, contemporary studies of well characterised longitudinal cohorts, including well-nourished control children, using modern immunological techniques, would help to understand better the immunology of stunting.

### Cognition and behaviour

In the medium-term, the cognitive, educational and behavioural components of the stunting syndrome impact child development.^3^ Stunting is one of the major risk factors, together with inadequate cognitive stimulation, iodine deficiency and iron-deficiency anaemia, for failure to attain full developmental potential.^227^ Stunted children have impaired behavioural development in early life,^228^ are less likely to enroll at school^229^ or enroll late,^230^ tend to achieve lower grades^231^ and have poorer cognitive ability^232^–^235^ than non-stunted children. Furthermore, stunted children are more apathetic, display less exploratory behaviour^236^ and have altered physiological arousal.^237^ Stunted children followed longitudinally in Jamaica were found to have more anxiety and depression and lower self-esteem than non-stunted children at age 17, after adjusting for age, gender and social background variables.^238^

Undernutrition affects areas of the brain involved in cognition,^239^ memory^240^ and locomotor skills.^241^ The brain has major energy demands in early childhood and most cerebral growth occurs in the first 2 years of life. However, the associations between poor linear growth and impaired neurodevelopment are not well understood.^239^ Whether there are defects in myelination, establishment of neural pathways or synaptic proliferation and subsequent pruning, or evidence for neuroinflammation is not clear, but field studies employing more sophisticated measures of brain structure and function are underway. Furthermore, malnutrition, micronutrient deficiencies (especially iron), recurrent infections, apathy, reduced exploration, poverty, low maternal education and decreased stimulation often co-exist in the same household, and all are likely to affect child development.^242^

The window of opportunity for improving cognitive outcomes remains uncertain. In long-term follow-up of a Guatemalan trial, individuals randomised to energy/protein supplementation had a 10% improvement in non-verbal cognitive ability, but only if supplements were given in the first 2–3 years of life.^243^ In the COHORTS study, growth in the first 2 years of life, but not later, was associated with higher school grades among adults.^255^ However, among children in the Young Lives study, those who caught up with physical growth after 8 years of age had improved cognitive testing scores compared with those who remained stunted;^216^ in Peru, these cognitive testing scores were very similar to those of children who had never been stunted.^245^ In the same Peruvian cohort, current rather than previous stunting was the more important factor associated with cognitive skills at school entry,^246^ and in Filipino children, change in HAZ from 2 to 11 years was associated with cognitive ability at 11 years.^247^ Taken together, studies suggest potential for catch-up in cognition, although the greatest improvements may be in those receiving interventions in early life before trajectories have been firmly established.^227^

### Long-term health and disease

Children who become stunted between conception and 2 years of age are at greater risk of poor health and lower socio-economic attainment throughout their lifetime. The excess risk of infectious morbidity and mortality apparent during childhood extends into adulthood.^248^ Stunted children lose ∼3.2 cm in adult stature for each decrement in HAZ at age 2 years;^249^ these negative impacts on stature (which translate into physical stamina) and cognition result in lower economic productivity, earning 8–46% lower wages^249^,^250^ and owning up to 66% fewer assets.^251^ Additionally, these effects are intergenerational: low birthweight is more common among infants whose mothers and even grandmothers were themselves stunted during early childhood.^249^ For Africa and Asia where 36% and 27% of children are stunted, respectively,^1^ these socio-economic consequences have a profound impact on the developmental capacity of entire societies.

Paradoxically, the metabolic syndrome, usually associated with over-nutrition, is more common in adults who were stunted in early childhood compared to those who experienced normal child growth.^249^ The ‘Developmental Origins of Health and Disease’ hypothesis proposes that nutritional deprivation during fetal or infant life^252^–^254^ triggers permanent epigenetic changes in metabolism (e.g. of lipids^253^ and glucose^252^) and organ anatomy and function (e.g. of blood vessels, liver and kidney^255^–^257^). While these changes may provide some survival benefit during fetal life by diverting nutrients away from growth to preserve vital functions, these same changes result in heightened risks of hypertension, cardiovascular disease, and type 2 diabetes, especially when aggravated by rapid weight gain and obesity after age 2 years. Accordingly, populations who have undergone a rapid transition from poverty and food insecurity to abundant access to a high-energy Western diet, such as India, are experiencing epidemics of diabetes and coronary heart disease.^258^ Conversely, in The Gambia, where socio-economic growth has been modest, little association between child stunting and adult metabolic syndrome has been detected.^259^

Observational data from birth cohorts indicate these associations between child stunting and adult disease are complex. Reviews of studies conducted primarily among high-income populations report that low birthweight is associated with elevated blood pressure during adulthood.^260^,^261^ Similarly, in pooled analyses of five birth cohorts from low- and middle-income countries,^244^,^249^ birthweight was inversely associated with adult blood pressure levels in analyses adjusting for adult BMI and height;^249^ but rapid (i.e. ‘catch-up’) linear growth between birth and mid-childhood^244^ was positively associated with elevated systolic blood pressure during adulthood. In these same cohorts, higher birthweight^249^ and catch-up rates of linear and ponderal growth between birth and mid-childhood^244^ were also associated with adult overweight. Importantly, the adverse associations with better linear growth were modest while increases in schooling, adult stature and wealth were substantial. Accordingly, increase in birthweight and linear growth velocity between conception and 2 years of age in children in developing countries is likely to reduce morbidity and mortality and provide substantial increases in human capital, with only modest trade-offs.

## POLICY AND PROGRAMME IMPLICATIONS

By current estimates, stunting prevalence is likely decline to 20% (or 127 million children) by 2025, which is some way off the World Health Assembly target.^2^ The current evidence base for interventions to improve maternal and child undernutrition has been comprehensively reviewed as part of the recent Lancet Nutrition series.^86^ These authors estimated that if a package of nutrition-specific interventions (provision of maternal folic acid, calcium, multiple micronutrients and balanced energy protein supplementation; promotion of breastfeeding and appropriate complementary feeding; management of moderate and severe acute malnutrition and preventive zinc and vitamin A supplementation) was scaled up to 90% coverage, stunting would be reduced by mean 20.3% (range 10.2–28.9%) and under-5 mortality would be reduced by 15% (range 9–19%).^86^

Most strategies targeting MDG 1 are health-related interventions focused on the immediate determinants of undernutrition.^262^ Trends in stunting reduction are uneven within regions, or even within countries;^263^,^264^ modelling suggests that an equity-based approach targeting the poorest and most marginalised communities would be more cost-effective and lead to more rapid declines in stunting prevalence.^265^ Many countries require policies that tackle the ‘dual burden’ of stunting and overweight that is emerging during the nutrition transition.^266^ Multi-sectoral approaches which combine nutrition-sensitive with nutrition-specific interventions are likely to have a greater impact on reducing stunting.^267^–^269^ For example, the Millennium Villages project in which simultaneous investments were made in agriculture, the environment, business, education, infrastructure and health showed reductions in stunting (from 36% to 28% among children <2 years) across nine countries after 3 years.^268^ In addition, an enabling environment needs to be built to address more distal factors causing stunting;^2^ these constitute long-term developmental goals that underlie the impressive reductions in stunting seen in some countries.^270^ In Brazil, for example, stunting declined from 37% in 1974–5 to 7% in 2006–7 following economic growth and reduced disparity, increased urbanisation, improved female education, decreased fertility rates, improved WASH and health service reform.^271^ Whilst awaiting such long-term national development, programmes should focus on implementing packages of evidence-based, multi-sectoral interventions which cover the life-course to achieve the intergenerational investment in human capital that stunting reduction could provide.

## DISCLAIMER STATEMENTS

**Contributors** AJP wrote the first draft of the manuscript, which was critically revised by JHH.

**Funding** AJP is funded by the Wellcome Trust (093768/Z/10/Z).

**Conflicts of interest** AJP and JHH are both investigators on the SHINE trial.

**Ethics approval** None.
